# Mathematical model of early Reelin-induced Src family kinase-mediated signaling

**DOI:** 10.1371/journal.pone.0186927

**Published:** 2017-10-19

**Authors:** Helge Hass, Friederike Kipkeew, Aziz Gauhar, Elisabeth Bouché, Petra May, Jens Timmer, Hans H. Bock

**Affiliations:** 1 Institute of Physics, University of Freiburg, Freiburg, Germany; 2 Clinic of Gastroenterology, Hepatology and Infectiology, Heinrich-Heine University Düsseldorf, Düsseldorf, Germany; 3 Centre for Neuroscience, University of Freiburg, Freiburg, Germany; 4 BIOSS Centre for Biological Signaling Studies, University of Freiburg, Freiburg, Germany; Wayne State University, UNITED STATES

## Abstract

Reelin is a large glycoprotein with a dual role in the mammalian brain. It regulates the positioning and differentiation of postmitotic neurons during brain development and modulates neurotransmission and memory formation in the adult brain. Alterations in the Reelin signaling pathway have been described in different psychiatric disorders. Reelin mainly signals by binding to the lipoprotein receptors Vldlr and ApoER2, which induces tyrosine phosphorylation of the adaptor protein Dab1 mediated by Src family kinases (SFKs). In turn, phosphorylated Dab1 activates downstream signaling cascades, including PI3-kinase-dependent signaling. In this work, a mechanistic model based on ordinary differential equations was built to model early dynamics of the Reelin-mediated signaling cascade. Mechanistic models are frequently used to disentangle the highly complex mechanisms underlying cellular processes and obtain new biological insights. The model was calibrated on time-resolved data and a dose-response measurement of protein concentrations measured in cortical neurons treated with Reelin. It focusses on the interplay between Dab1 and SFKs with a special emphasis on the tyrosine phosphorylation of Dab1, and their role for the regulation of Reelin-induced signaling. Model selection was performed on different model structures and a comprehensive mechanistic model of the early Reelin signaling cascade is provided in this work. It emphasizes the importance of Reelin-induced lipoprotein receptor clustering for SFK-mediated Dab1 trans-phosphorylation and does not require co-receptors to describe the measured data. The model is freely available within the open-source framework Data2Dynamics (www.data2dynamics.org). It can be used to generate predictions that can be validated experimentally, and provides a platform for model extensions both to downstream targets such as transcription factors and interactions with other transmembrane proteins and neuronal signaling pathways.

## Introduction

Reelin plays a pivotal role in brain development. During embryonic development, Reelin is secreted by Cajal-Retzius cells, a specialized cell population in the developing central nervous system (CNS), which coordinates migration of postmitotic neurons in laminar structures of the brain like hippocampus, neocortex and cerebellum. The autosomal recessive mutant mouse reeler [1] underlines the importance of Reelin. Affected mice are characterized by ataxic behavior and display multiple defects in the CNS such as cerebellar hypoplasia and impaired cortical lamination [1]. Furthermore, an association between altered Reelin expression/signaling and several psychiatric and neurological disorders including Alzheimer disease, autism, major depression, schizophrenia, and temporal lobe epilepsy has been demonstrated [2–5]. However, the precise contribution of Reelin signaling events in this context remains unclear.

The most important transmembrane receptors for Reelin are the Very-low-density lipoprotein receptor (Vldlr) and Apolipoprotein E receptor 2 (ApoER2), reviewed in [6,7]. By binding to these receptors, Reelin induces tyrosine phosphorylation of the intracellular adapter protein Disabled-1 (Dab1) by non-receptor tyrosine kinases of the Src family (SFKs) [8–10]. A deficiency in Dab1, Reelin, or both ApoER2 and Vldlr in mice causes indistinguishable reeler phenotypes [11–15]. It has been shown that Reelin does not only bind to ApoE receptors, but also to other transmembrane proteins e.g. integrin *α*3*β*1 [16,17], Eph and ephrin proteins [18,19] and amyloid precursor protein (APP) [20]. As Dab1 is also interacting with the intracellular domains of different transmembrane proteins [21–26], a complex network of potential signaling interactions is imaginable. The precise regulation of this network for context-dependent biological functions of Reelin in the CNS and non-neural tissues has widely remained unclear.

Data-based modeling of signal transduction networks has emerged as a new approach to gain insight into cellular information processing and its perturbation in pathologies [27–29]. In this work, a mechanistic model based on time-resolved measurements of the early dynamics of the Reelin signaling pathway is developed [30,31]. Hypotheses about cluster formation of lipoprotein receptors after binding to Reelin and the interplay between SFKs and Dab1 phosphorylation are evaluated via model selection, including the role of feedback mechanisms to terminate Reelin-induced signaling. Model reduction was performed to assure identifiable parameters, and thus finite prediction uncertainties for experiment-guided model extensions or connection of model predictions to defects associated with Reelin-mediated signaling [1,2,6,32].

## Materials and methods

### Ethics statement

NMRI wild-type mice were obtained from Janvier. Vldlr and ApoER2 knockout mice and wildtype littermates were housed in the local breeding facility (ZETT, Zentrale Einrichtung für Tierforschung und Tierschutzaufgaben, Heinrich-Heine-University Düsseldorf). All animal applications were reviewed and approved by the appropriate authorities (O62/13) and were performed in accordance with the German animal protection law.

### Preparation and treatment of primary cortical neuron cultures

Cortical neurons were isolated from E15.5 NMRI wild-type mice, with E0.5 defined as the morning of plug detection, and cultured as described in [18]. Cerebral cortices were dissected in Hanks' Balanced Salt Solution (HBSS) (Life Technologies) and trypsinized with 0.05% trypsin-EDTA (Life Technologies) at 37°C for 25 min. Trypsinization was stopped by addition of an equal amount of fetal calf serum (FCS) and cells were triturated and centrifuged at 500x g for 5 min. The cell pellet was resuspended in DMEM (4.5 g/l glucose) supplemented with 8% FCS and 1x10^6^ cells/ well were plated on 6-well plastic cell culture dishes coated with 0.05 mg/ml poly-D-lysine (Sigma). After 18 h the medium was replaced by serum-free Neurobasal medium supplemented with 2% B27 supplement and 1 mM GlutaMax (Life Technologies). Neurons were cultured for 5 days at 37°C and 5% CO2 and then stimulated with Reelin or control-conditioned supernatant as described in [18] and analyzed by Western blotting. Src-family kinase inhibitor PP2 (10 μM) (Tocris Bioscience, 1407) and negative control PP3 (10 μM) (Tocris Bioscience, 2794) were added to the culture medium 30 min prior to each Reelin stimulation time point.

### Quantification of Reelin

**Fig 1:**
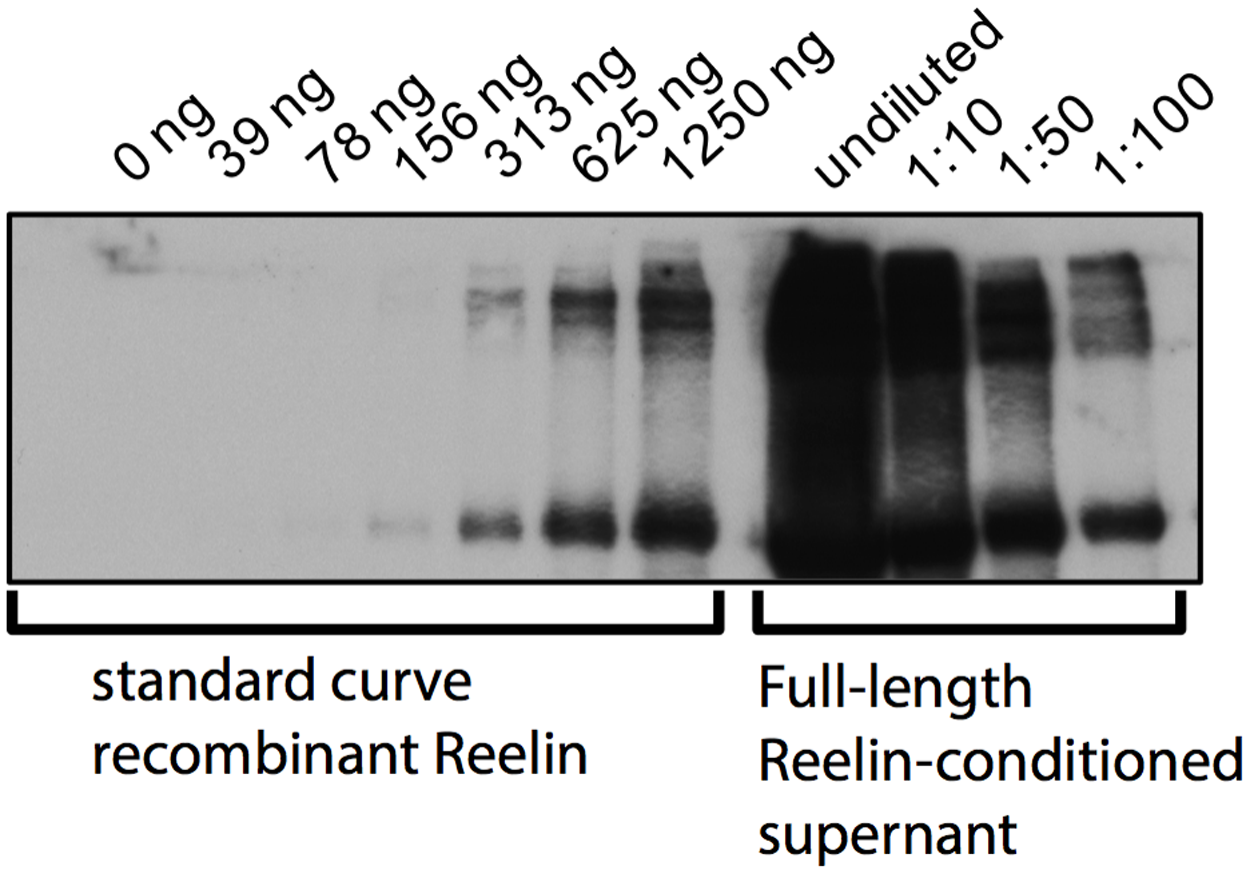
Quantification of the absolute Reelin amount in supernatants used for stimulation. Standard curve of recombinant Reelin protein and several dilutions of Reelin-conditioned supernatant, measured by Western blotting. Lanes ‘undiluted’ and ‘1:10’ were omitted from calculation. Measurements comprise three biological replicates.

For Reelin stimulation of neurons, full-length Reelin or control-conditioned supernatant (Mock) from stably transfected HEK-293 was used. To quantify the absolute amount of Reelin protein in conditioned supernatants, a standard curve of recombinant Reelin protein was utilized. To minimize inaccuracies due to the amount of loaded sample e.g., several dilutions of the Reelin-conditioned supernatant were examined. As a result, a concentration of 330ng/μl Reelin was used for stimulation experiments (cf. Fig 1).

### Western blotting

**Fig 2:**
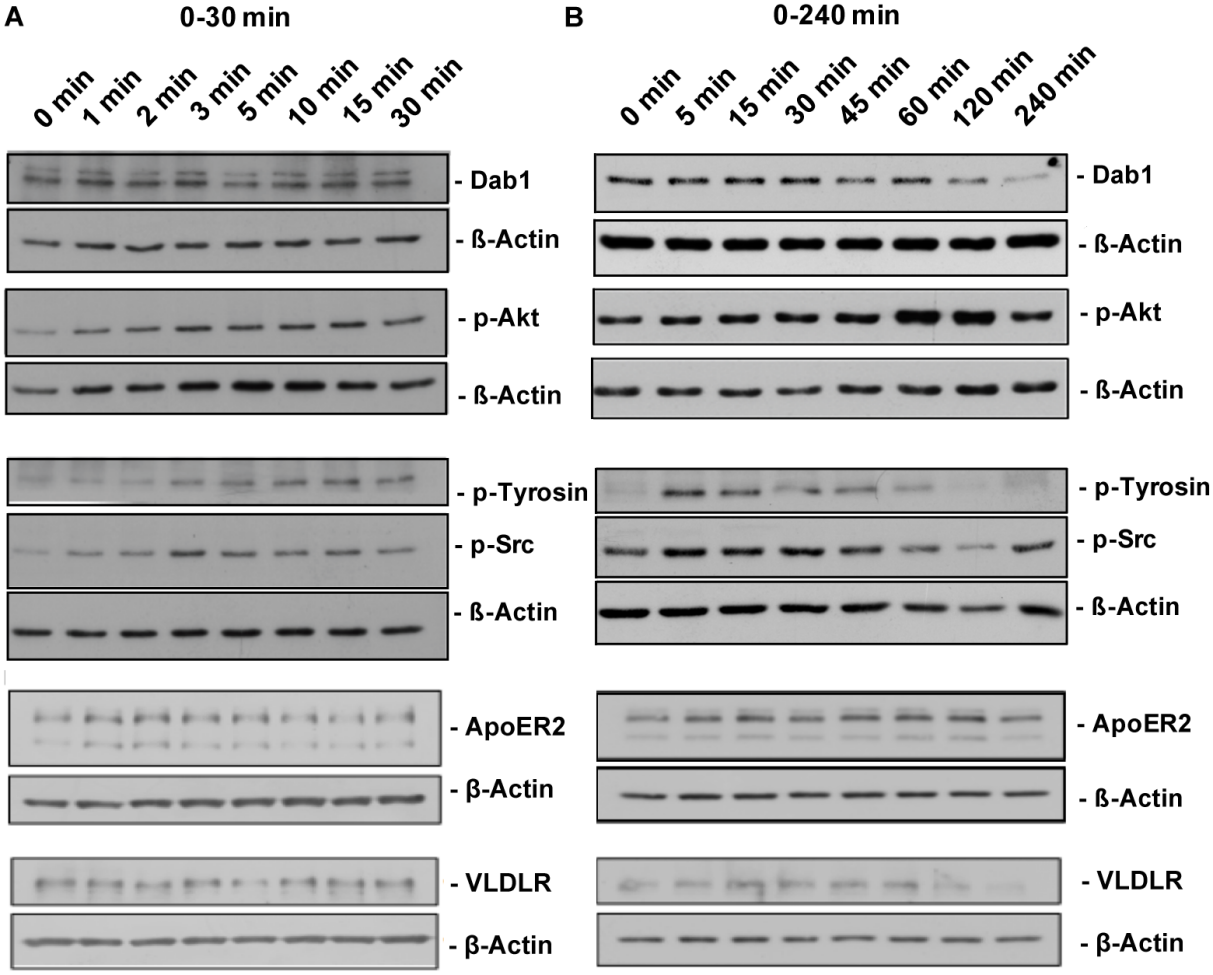
Time course of Reelin stimulation in neurons. Activation and degradation of core components of lipoprotein receptor-dependent Reelin signaling were measured by use of quantitative western blotting. Representative blots of tyrosine-phosphorylation of the neuronal adaptor protein Dab1, total Dab1, total VLDLR and total ApoER2, activation of Src family kinases as well as activation of the PI3 kinase targeting molecule Akt/PKB in primary cortical neurons prepared from E15.5 mice are shown for (A) 0–30 min and (B) 0–240 min of Reelin stimulation.

Cell lysates from neuron cultures were harvested in RIPA buffer (50 mM Tris-HCl pH 8.0, 1% NP40, 150 mM NaCl, 0.1% SDS, 1 mM EDTA pH 8.0, 12 mM sodium deoxycholate) supplemented with protease inhibitor (cOmplete ULTRA Tablets—Mini, EDTA-free, Roche) and phosphatase inhibitor (Phosphatase Inhibitor Cocktail 2 and 3, Sigma). Cell debris was removed from lysates by centrifugation (30 min at 17982x *g*, 4°C). Lysates were mixed with sample buffer (NuPAGE LDS Sample Buffer, Life Technologies) with 0.1 M DTT, heat-denatured, separated by SDS-PAGE and transferred to nitrocellulose membranes (GE Healthcare). For protein detection, membranes were incubated with the following primary antibodies: Mouse anti-ß-actin (1:5000, Abcam ab3280), rabbit anti-Dab1 (1:2000, Millipore MABS167), rabbit anti-p-Akt(S473) (1:1000, Cell Signaling 4060), rabbit anti-p-Src family (Y418) (1:1000, Cell Signaling 6943), mouse anti-p-tyrosine 4G10 (1:1000, Millipore 05–231), rabbit anti-Src (32G6) (Cell Signaling 2123, 1:1000), rabbit anti-Fyn (p59Fyn) (Epitomics 1562–1, 1:1000), rabbit Akt (pan) (C67E7) (Cell Signaling 4691, 1:1000), rabbit anti-ApoER2 (EPR3326) (Abcam ab108208, 1:1000), goat anti-Vldlr antibody (R&D systems AF2258, 1:500), mouse anti-Reelin G10 [33], 1:2000) and respective secondary HRP-coupled antibodies: anti-mouse IgG (GE Healthcare NA931V, 1:7500), anti-rabbit IgG (GE Healthcare NA934V, 1:7500) and anti-goat IgG (Santa Cruz sc-2020, 1:5000). Detection was accomplished by using enhanced chemiluminescence and Fuji Super RX films. Densitometric quantification was done with ImageJ. An exemplary measurement is shown in Fig 2.

### Mathematical modelling

Biochemical reaction networks are frequently described by mechanistic models via ordinary differential equations (ODEs) according to the laws of mass action. This allows for detailed description of the measured time courses and quantitative assessment of the underlying network, since in mechanistic models every model component has a corresponding biological counterpart. The time courses of their concentrations are calculated by solving the corresponding ODE system

$$\dot{x}\left(t\right)=f\left(x\left(t\right),\theta_{d}\right),$$ (1)

depending on initial values and kinetic rate parameters comprised in *θ_d_*. These are linked to measured concentrations of the involved proteins by an observational function

$$y\left(t\right)=g\left(x\left(t,\theta_{d}\right),\theta_{o}\right)+\epsilon \left(t\right),$$ (2)

with the assumption of Gaussian errors *ε* ∼ *N*(0, *σ*). In addition, the observational function includes scaling and offset parameters, summarized in *θ_o_*.

### Pre-processing of Western blot data

To obtain the data *y(t)* of Eq (2), a log-transformation of the measurements was performed, which typically results in Gaussian errors for immunoblot measurements [34]. Next, the Western blot data were pre-processed according to the data averaging method described in [35], performed in the open-source R package blotIt. It estimates the relative scaling for multiple biological replicates, which results in an average time-course and corresponding measurement uncertainties. In this context, biological replicates indicate independent experiments, e.g. cells from different mice or pipetted and stimulated in distinct wells. For time points *t*_*i*_, experimental conditions *c* and biological replicates *j*, the objective function

$$\chi^{2}\left(s_{j},y_{i,c}\right)=\sum_{i,c,j}{\left(\frac{\frac{y_{i,c}}{s_{j}}-Y_{i,c,j}}{\frac{y_{i,c}}{s_{j}}\;\sigma_{i,c,j}}\right)}^{2}$$ (3)

is minimized, which sums over all measurements Y_i,c,j_ [36]. Eq (3) estimates the systematic differences between distinct biological replicates by the replicate-dependent scaling factor *s*_*j*_, and allows to calibrate the mechanistic model to an average data set *y*_*i*,*c*_ with corresponding measurement error *σ*_*i*,*c*_ being the variance of the residuals of Eq (3).

### Calibration of the mechanistic model

To compare the model response to the pre-processed data, the scaled log-likelihood is calculated via

$$-2\;\text{log}\left(L\right)=\chi^{2}\left(\theta \right)=\sum_{i,c}{\left(\frac{y_{i,c}-g_{c}\left(x\left(t_{i},\theta_{d}\right),\theta_{o}\right)}{\sigma_{i,c}}\right)}^{2}+const.$$ (4)

for measurements at time points *t*_*i*_ and experimental condition *c*.

Within the maximum likelihood framework, the optimized parameter set $\hat{\theta }$ is estimated via minimization of *χ*^2^(*θ*), with *θ* comprising *θ*_*o*_ and *θ*_*d*_. Here, the parameters were estimated on the basis of time-resolved quantitative data of involved proteins and dose-response measurements at a specific time point.

To acquire parameter uncertainties in non-linear models, the profile likelihood approach is utilized [37,38]. Therein, the profile likelihood of parameter *θ*_*j*_ is given by

$$PL\left(\theta_{j}\right)={\text{min}}_{\theta_{j_{{}_{i\neq j}}}}\chi^{2}\left(\theta \right).$$ (5)

The confidence interval of parameter *θ_j_* is then given by all parameter values for which the corresponding likelihood value does not exceed the threshold given by $\chi_{1,\alpha }^{2}$, the distribution with one degree of freedom and confidence level *α*,

$$CI_{\theta_{j},\alpha }=\left\{\theta_{j}│PL\left(\theta_{j}\right)\leq \chi^{2}\left(\hat{\theta }\right)+icdf\left(\chi_{1.\alpha }^{2}\right)\right\}.$$ (6)

To assess the model's prediction uncertainty for the purpose of experimental validation, prediction bands were calculated. These were introduced for non-linear ODE models in [39] and in this case computed via fast integration methods [40].

## Results

In the following, a mechanistic dynamic model of the early Reelin signaling cascade is established. For that purpose, prior knowledge of Reelin-mediated signaling is translated into distinct mathematical models. Model selection will be performed from model variants reduced to the information content of the data, followed by thorough model validation.

### Model selection

**Fig 3:**
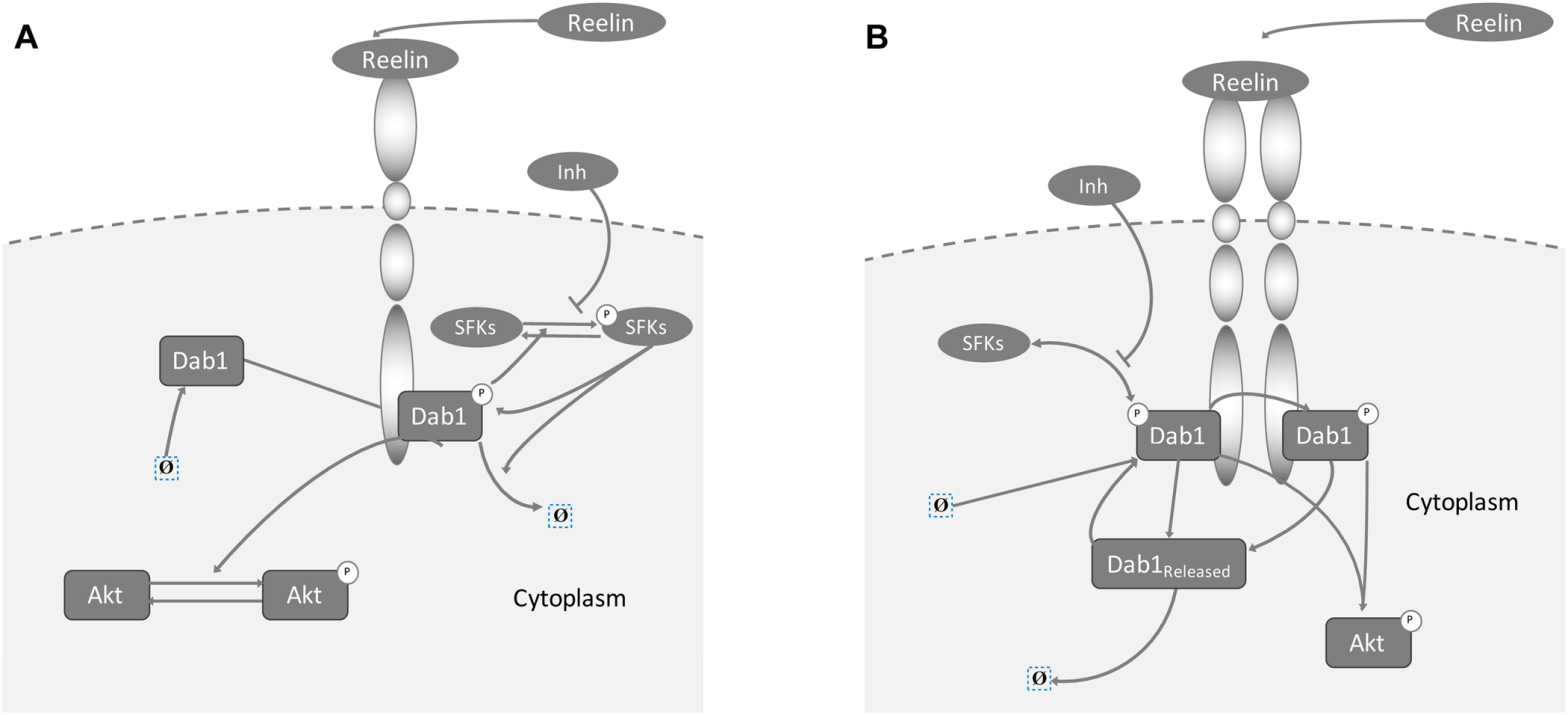
Scheme of the monomer (A) and complex (B) signaling models. In the monomer model variant (A), Dab1 is activated by tyrosine phosphorylation after binding of Reelin to the receptor. The initial phosphorylation of Dab1 initiates phosphorylation of SFKs, which results in a positive feedback. Subsequently, p-Dab1 induces activation of downstream targets, such as Akt. Signaling is negatively regulated by degradation of phosphorylated Dab1, which is further increased through the Reelin-dependent activation of SFKs. In the complex model (B), clusters of the lipoprotein receptors are formed. These bind the adaptor protein Dab1, which is phosphorylated by SFKs. As a feed-forward loop, SFKs activated in a p-Dab1/SFK complex can trans-phosphorylate other Dab1 proteins bound to the receptor complex. The pathway is regulated by ubiquitination and degradation of phosphorylated Dab1.

To obtain a mathematical model of Reelin-induced Dab1 and Src family kinase (SFK) activation, we performed model selection based on two different hypotheses. In the first model, defined as ‘monomer’ model (Fig 3A), binding of Reelin to its canonical receptors leads to tyrosine phosphorylation of Dab1 [41–43]. In this model, it is assumed that an initial phosphorylation of Dab1 initiates binding and phosphorylation of SFKs, which results in a positive feedback. Subsequently, Akt is activated downstream of tyrosine-phosphorylated Dab1 [43–45]. The signaling is terminated by degradation of phosphorylated Dab1, which is further increased through the positive feedback regulation of Dab1 tyrosine phosphorylation by activated SFKs [8,46–48]. In contrast, the second model variant, denoted ‘complex’ model, is based on mechanisms proposed in [42,49]. Here, SFKs function as crucial components initiating the Dab1 phosphorylation that are able to trans-phosphorylate other Dab1 proteins bound to clustered receptors. The receptor cluster is formed by Reelin stimulation (Fig 3B). Detailed information including model equations and utilized data sets, including the number of biological replicates, can be found in S1 Appendix.

**Fig 4:**
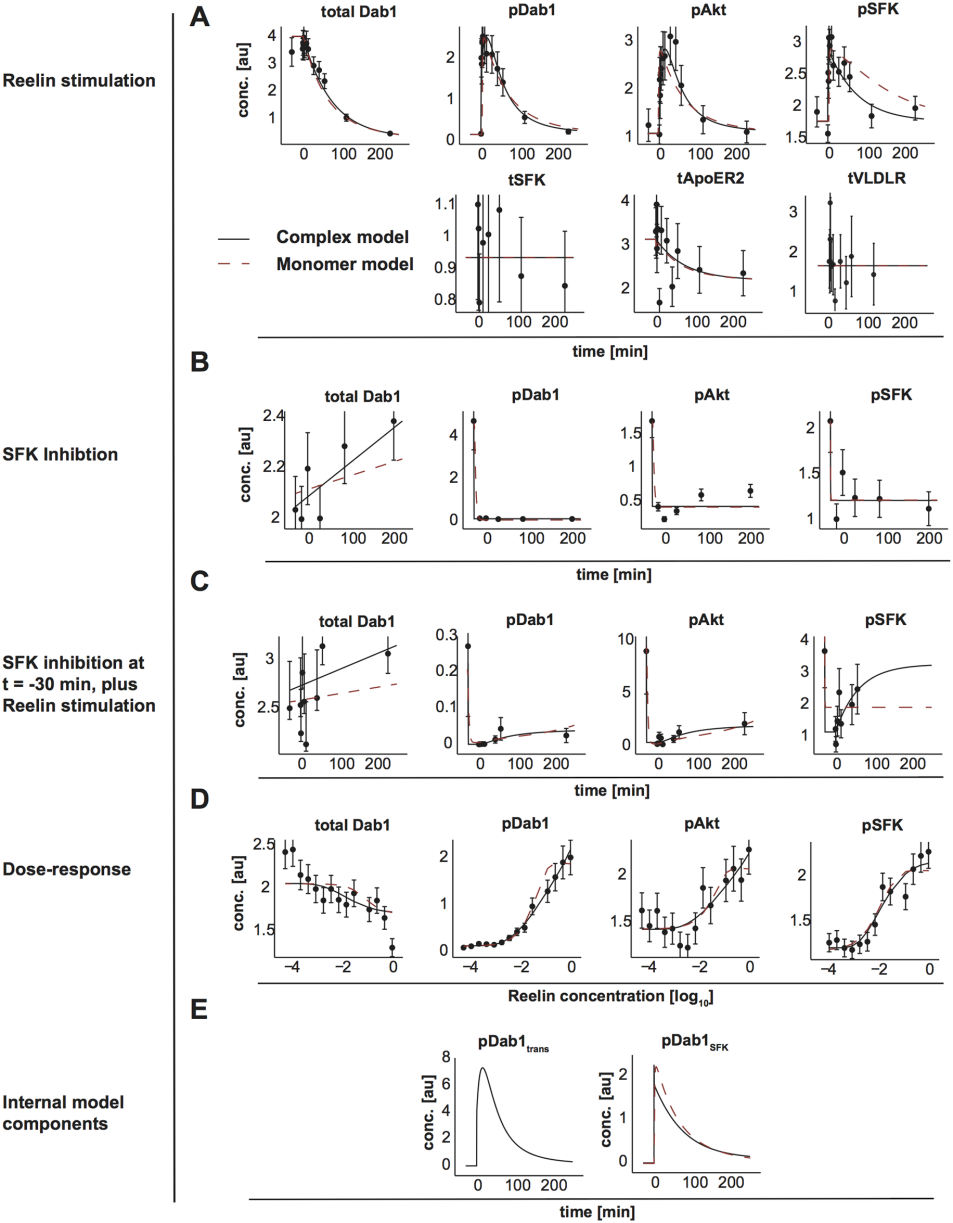
Model fits of the monomer and complex models. Data points are shown as black dots with corresponding uncertainty, the model trajectories as red dashed line (monomer model) or black solid line (complex model). (A) Data and model dynamics after stimulation with a saturating concentration of Reelin. (B) Data and model trajectories after pretreatment with a pan-SFK inhibitor. (C) Reelin stimulation after SFK inhibition for 30 minutes. (D) Data and model response for a dose-response measurement of Reelin at t = 10 min. Reelin concentration is shown in log-space on the x-axis in arbitrary units, with 0 corresponding to the saturating concentration. (E) Dynamics of the model components for phosphorylated Dab1 after trans-phosphorylation (pDab1_trans) or after direct phosphorylation succeeding SFK binding (pDab1_SFK_bound).–[au]: arbitrary units

Both model variants are able to describe the majority of the measurements (Fig 4). However, the assumed interplay of SFKs and Dab1 in the monomer model cannot fully reproduce the time courses of p-Dab1 and p-SFK, especially for Reelin stimulation with and without SFK inhibition (Fig 4A and 4C, red dashed lines). In addition, the p-Dab1 and p-AKT concentrations are overestimated for large doses of Reelin (Fig 4D). In contrast, the complex model is able to describe the time-resolved data obtained after Reelin stimulation as well as the dose-response data (Fig 4, black solid lines). Thereby, the parameters were estimated to achieve a fast but transient initial phosphorylation step of Dab1 with bound SFK, followed by trans-phosphorylation of the remaining Dab1 adapter proteins with delayed degradation (Fig 4E). Comparing both models, the complex model is substantially better than the monomer model variant, leading to a 13.0% improvement in the goodness of fit.

Model selection among a set of distinct model variants can be conducted via the Bayesian information criterion (BIC), defined by

$$BIC=\chi^{2}\left(\hat{\theta }\right)+\text{ln}\left(n\right)*p$$ (7)

Therein, χ^2^ is taken from Eq (4), *n* denotes the number of data points and *p* the number of parameters in each model, respectively [50]. Since the BIC penalizes the number of parameters and assumes asymptotic properties of the likelihood, it is beneficial if the model variants are tailored to the amount of information available in the experimental data [51]. In addition, these models feature finite prediction confidence intervals and allow for experimental validation (see Section Model validation). Calculation of the BIC for both reduced models yields a difference of 14.44 (for details see S1 Appendix), which poses strong evidence against the monomer model [52].

### Model reduction

Model reduction was performed via parameter profile likelihood (see Eq (5)). Thereby, we could reduce the model whenever a parameter could be driven to plus or minus infinity without impairing the model's likelihood above a statistically significant level [51]. In the following, the model reduction of the complex model is briefly outlined.

**Fig 5:**
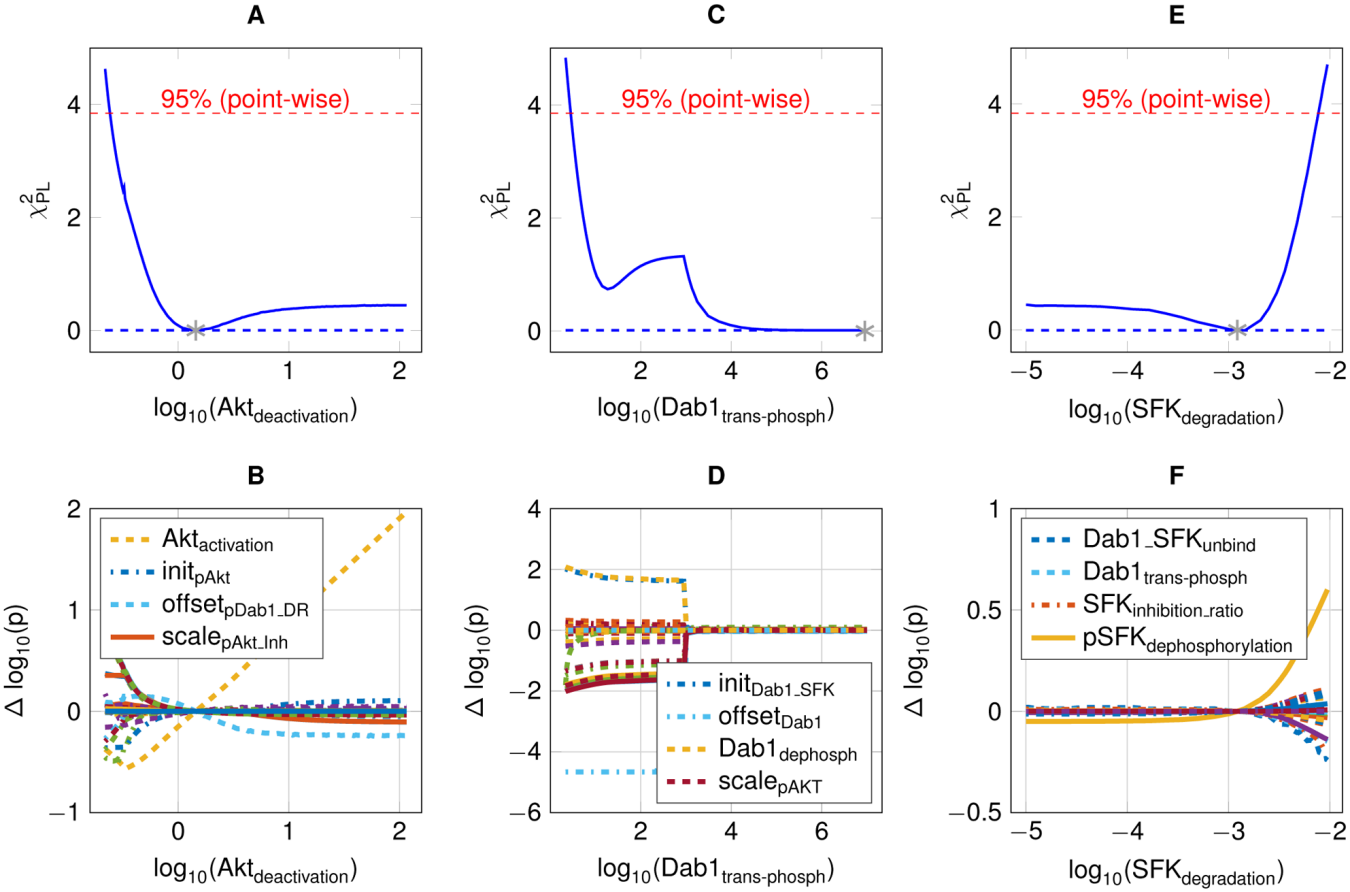
Overview of model reduction steps. (A) Parameter profile likelihood of the Akt deactivation. (B) Dependency of Akt activation and deactivation leading to a coupling with phosphorylated Dab1. (C) Parameter profile of Dab1 trans-phosphorylation that does not exceed the 95% threshold for high values. (D) Remaining model parameters are unchanged in the upper limit. (E) Parameter profile of SFK degradation, which is non-identifiable towards zero. (F) Remaining model parameters re-optimized in turn.

Analyzing the profile of Akt deactivation via dephosphorylation, the parameter *Akt*_*deact*_ does not exceed the 95% threshold for large values (Fig 5A), with positive relation to the Akt activation (Fig 5B). As described in [53], both parameters reaching infinity will result in an Akt time course that parallels the time course of the upstream signaling event, in this case tyrosine phoshorylated Dab1. Thus, the model reduction leads to a functional relation by *pAkt* = *scale*_*pAkt*_ *pDab*1. Also, the time-scale of Dab1 trans-phosphorylation can become infinitely fast, as the profile of the corresponding profile towards infinity suggests (Fig 5C and 5D). This behavior is congruent with the known fast trans-phosphorylation of receptors [54]. As a result, the plateau-like time course of phosphorylated Dab1 is steered by its interaction with SFKs in relation to ubiquitin-dependent p-Dab1 degradation. In addition, the profile of SFKs degradation does not exceed the 95% threshold for parameter values going to minus infinity (Fig 5E). Moreover, no other model parameter is adjusted in the limiting case (Fig 5F). Thus, SFK internalization with subsequent degradation or dephosphorylation is not needed to describe the available data and can be subsumed in a single deactivation step, compatible with the roughly constant measurements of total SFK (Fig 4A). In conclusion, the reduced model possesses only finite parameter profiles (S1 Fig).

**Fig 6:**
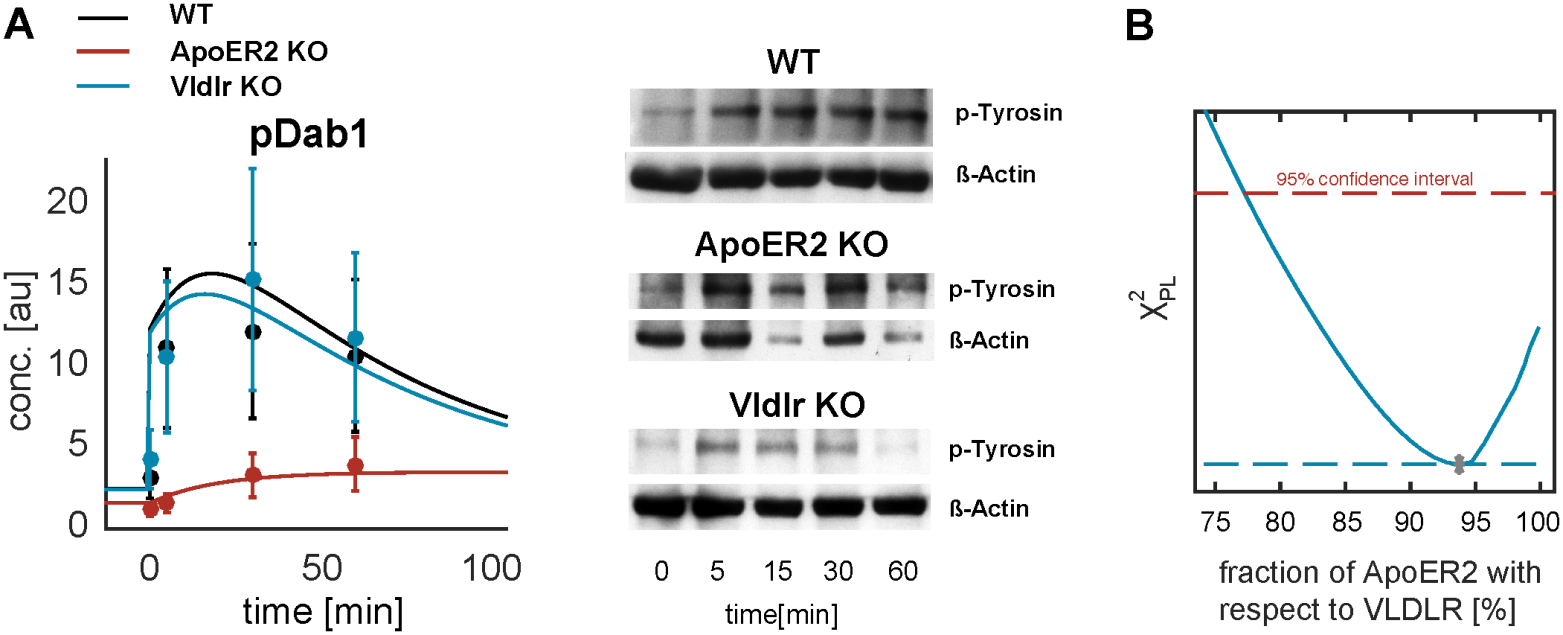
Time-course of tyrosine-phosphorylated Dab1 after Reelin stimulation in neurons from WT, Vldlr -/- and ApoER2 -/- knockout mice, measured on a single Western blot. (A) Data is shown as dots with their respective error bars from N = 4 biological replicates. Model trajectories as lines from the complex model that is calibrated on all available data. Representative Westernblots for all conditions are shown on the right. Detailed information about the knockout mice and antibodies are given in Section Materials and Methods. (B) Parameter profile of the amount of ApoER2 within the sum of all receptors, which is compatible with 100% denoting that no Vldl receptors need to be involved in the signaling.

For model calibration and selection, only one type of receptor was implemented in the mechanistic models. Yet, distinct functions of both high-affinity Reelin receptors Vldlr and ApoER2 were reported in the literature [6,14,55], resulting in a reeler-like phenotype only after combined inactivation of both receptors in mice. Thus, we performed time-course measurements of tyrosine-phosphorylated Dab1 on a single Western blot, with neurons from either WT, Vldlr -/- or ApoER2 -/- mice. Two distinct receptors were included in the mechanistic model, with potentially different kinetic rates, and parameters were estimated on all available data. The resulting model fit was able to describe the measurements (Fig 6A). The parameter profile corresponding to the amount of ApoER2 within all receptors was compatible with 100% (Fig 6B). In addition, the trans-phosphorylation parameter of Dab1 bound to Vldl receptors was compatible with 0, which can be explained by the slow phosphorylation of Dab1 measured in the ApoER2 -/- mice that only required slow activation of receptor monomers to describe the measurements. To conclude, the impact of signal transduction via Vldlr on the short time-scale in mouse embryos was not significant within our experiments. Thus, we assumed a single high-affinity receptor in our models of the early Reelin-mediated signaling. Yet, this simplification may not hold for a prolonged time frame and differing experimental conditions, or if the model is extended to include other targets, e.g. intersectin-1.

### Model validation

**Fig 7:**
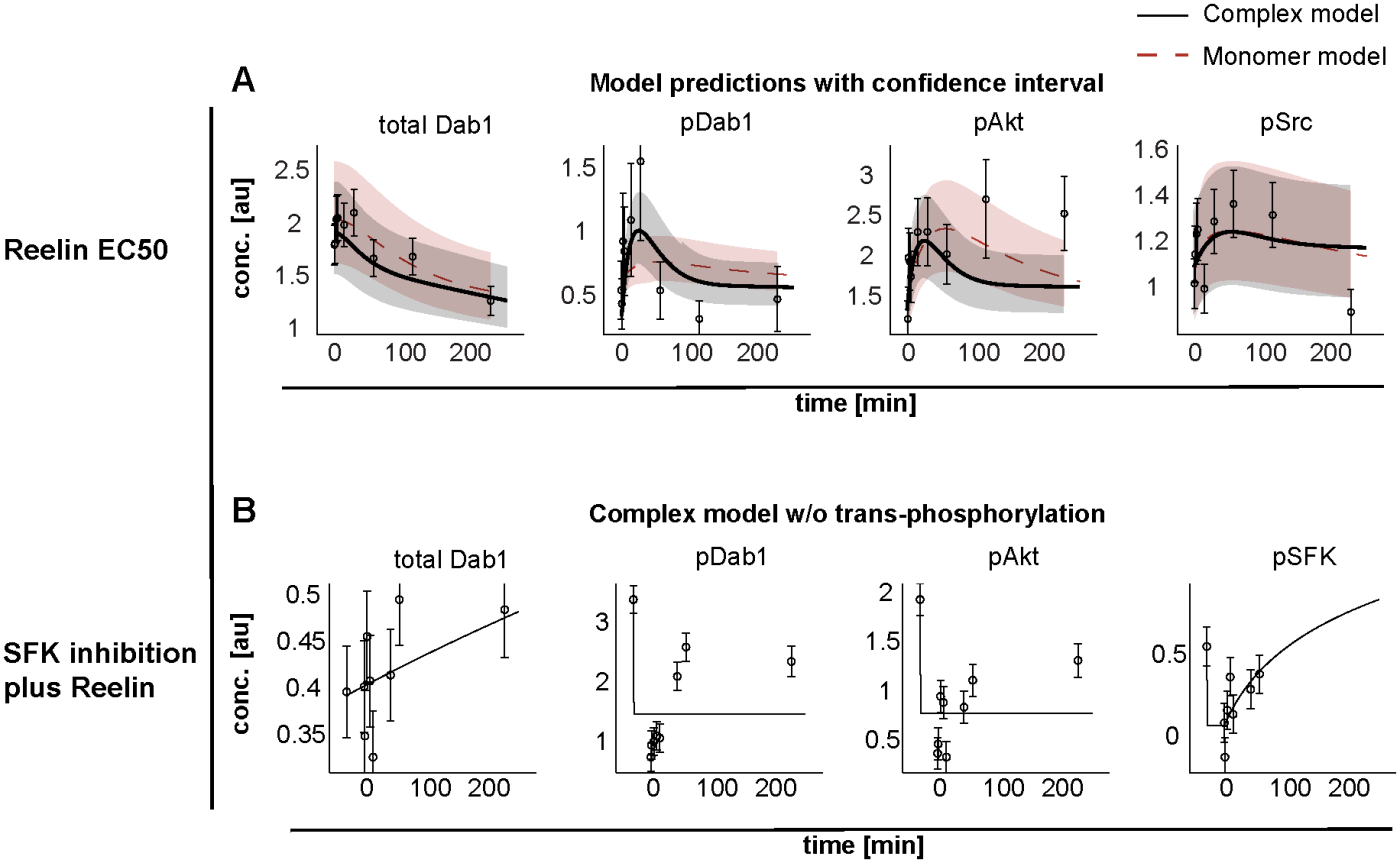
Model validation. (A) Prediction bands for an EC50 concentration of Reelin for both monomer (red dashed lines) and complex (black solid lines) models. Data is shown as open circles with errors. The shaded areas correspond to the standard deviation of the model predictions. (B) Model trajectories for Reelin stimulus after SFK inhibition, if SFK-dependent trans-phosphorylation between Dab1 proteins in the cluster is prohibited.

To further validate the selected model, we measured time-course data at the half maximal effective dose (EC50) of Reelin, determined from the dose-response of p-Dab1, but did not use the data for parameter estimation. Instead, the data was compared to the predictions of both monomer and complex model (Fig 7A). The validation data reinforced the hypothesis of the complex model, since the monomer model is not able to capture the transient activation of p-Dab1.

The importance of Dab1 trans-phosphorylation within the receptor complex is emphasized if the model is deprived of this capability, leading to a significantly worse description of the data (Fig 7B). The likelihood-ratio test rejects the exclusion of trans-phosphorylation of Dab1 via SFKs with a p-value of 6.60 * 10^−8^. In addition, the reduced mathematical model allows to test the hypothesis of a crucial co-receptor [19]. For the available dose-response data no co-receptor is needed, since it is well described by the model with goodness-of-fit of 1.03 (Fig 4D).

## Discussion

In this work, we performed a comprehensive model development focusing on early Reelin-induced and SFK-mediated signal transduction. Time-resolved quantitative Western blot measurements in cortical neurons of mice were exploited to select between two different model hypotheses. These comprised either Dab1 phosphorylation in receptor monomers, amplified by SFKs, or a SFK-mediated trans-phosphorylation of Dab1 that is bound to a receptor complex formed after Reelin stimulation. Only one type of high-affinity Reelin lipoprotein receptor was assumed, supported by measurements in VLDLR -/- and ApoER2 -/- mice. Model reduction based on the method of profile likelihood led to identifiable models featuring finite prediction intervals. Through interplay of experimental design and model validation, measurements with Reelin stimulation after pan-SFK pre-inhibition as well as with Reelin stimulation using the EC50 concentration [56] were conducted, and confirmed that clusters of Dab1 adaptor proteins, brought into close proximity by lipoprotein receptors after Reelin stimulation, and subsequently trans-phosphorylated through SFKs [42,49] are required to describe the effects seen in the data.

The fast, receptor tyrosine kinase-like activation of Dab1 followed by a prolonged plateau in its phosphorylation for high doses of Reelin as well as a transient activation for an EC50 dose is well described by the postulated model structure. Therein, SFKs could be confirmed as crucial part of the network, leading to an almost complete absence of Dab1 phosphorylation after pan-SFK inhibition, which persisted to a great extend after Reelin stimulation [45,57]. Moreover, activation of Akt depends linearly on the amount of tyrosine-phosphorylated Dab1. Regulation of the Reelin signaling cascade functions through degradation of Dab1 independent from SFKs turnover [58,59].

Concerning the requirement of possible co-receptors for activation of Reelin-dependent Dab1 signaling, data from dose-response measurements were analyzed. These revealed that the behavior of both tyrosine-phosphorylated Dab1 as well as Akt and SFK phosphorylation for Reelin stimulations spanning four orders of magnitude are well described by a model without any co-receptor. Thus, either the hypothesized component [19,60] is not limiting within the concentration span of our measurements, or ApoE receptors and SFKs alone are sufficient for Dab1-related signaling [61].

In the last decade, multiple hypotheses arose considering the details of Dab1 tyrosine phosphorylation and their limiting factors [9,19,47–49,57,60,62]. These were mostly answered through extensive data obtained e.g. by Western blotting and mice bearing gene modifications. On the contrary, ODE based modeling is frequently used to unravel dynamical features from signaling pathways, whereby predictions are used to validate and build up confidence in the model [30,31]. Through iterations between data analysis and experimental measurements, we could establish a mechanistic model that is able to reproduce the data and allows for predictions with finite confidence intervals, which could be verified for the case of an altered Reelin concentration. This demonstrates the power of time-course measurements and mathematical modeling in the understanding of signaling pathways.

Yet, it is important to formulate the model in a way that the number of its equations and parameters are appropriate for the information given in the data, and the balance between describing the dynamics of the measurements and a fully identifiable parameter set is crucial. Moreover, applicability of the model for distinct predictions and model extensions have to be validated carefully, since model development based on the method of profile likelihood is purely data-driven and solely reflects the analyzed biological setting [37,51]. In conclusion, a comprehensive mechanistic model describing the early Reelin signaling cascade through Dab1 and SFKs with subsequent Akt activation as an example for a p-Dab1-dependent downstream target is provided in this work. Therein, hypotheses of a vital interplay between SFKs and Dab1 as well as clustering of lipoprotein receptors could be confirmed. Since the complexity of the model is tailored to the information content of the data, finite prediction confidence intervals can be generated in order to guide experimental design and future model extensions, such as interactions of p-Dab1 with CULLIN and SOCS for proteasomal degradation. Also, activation of small GTPases or members of the CRK family as well as crosstalk with NMDA receptors can be implemented to shed light on the role of Reelin in neuronal positioning, dendrite outgrowth, modulation of synaptic plasticity and its role in neurodegenerative diseases.

## Supporting information

S1 Fig Parameter profiles of all model parameters of the complex model. Likelihood profiles of kinetic and observational parameters for all conditions after model reduction, which reach the 95% threshold in both directions, thus are identifiable.
(PDF)

S1 Appendix Mathematical model of Reelin signal transduction. (PDF)
