# Supplementary material for: Mathematical model of early Reelin-induced Src family kinase-mediated signaling
Source: PLoS One. 2017 Oct 19;12(10):e0186927. doi: 10.1371/journal.pone.0186927 (PMC5648249; doi:10.1371/journal.pone.0186927)
Supplement: S1 Fig — Likelihood profiles of kinetic and observational parameters for all conditions after model reduction, which reach the 95% threshold in both directions, thus are identifiable. (PDF) [file pone.0186927.s001.pdf]

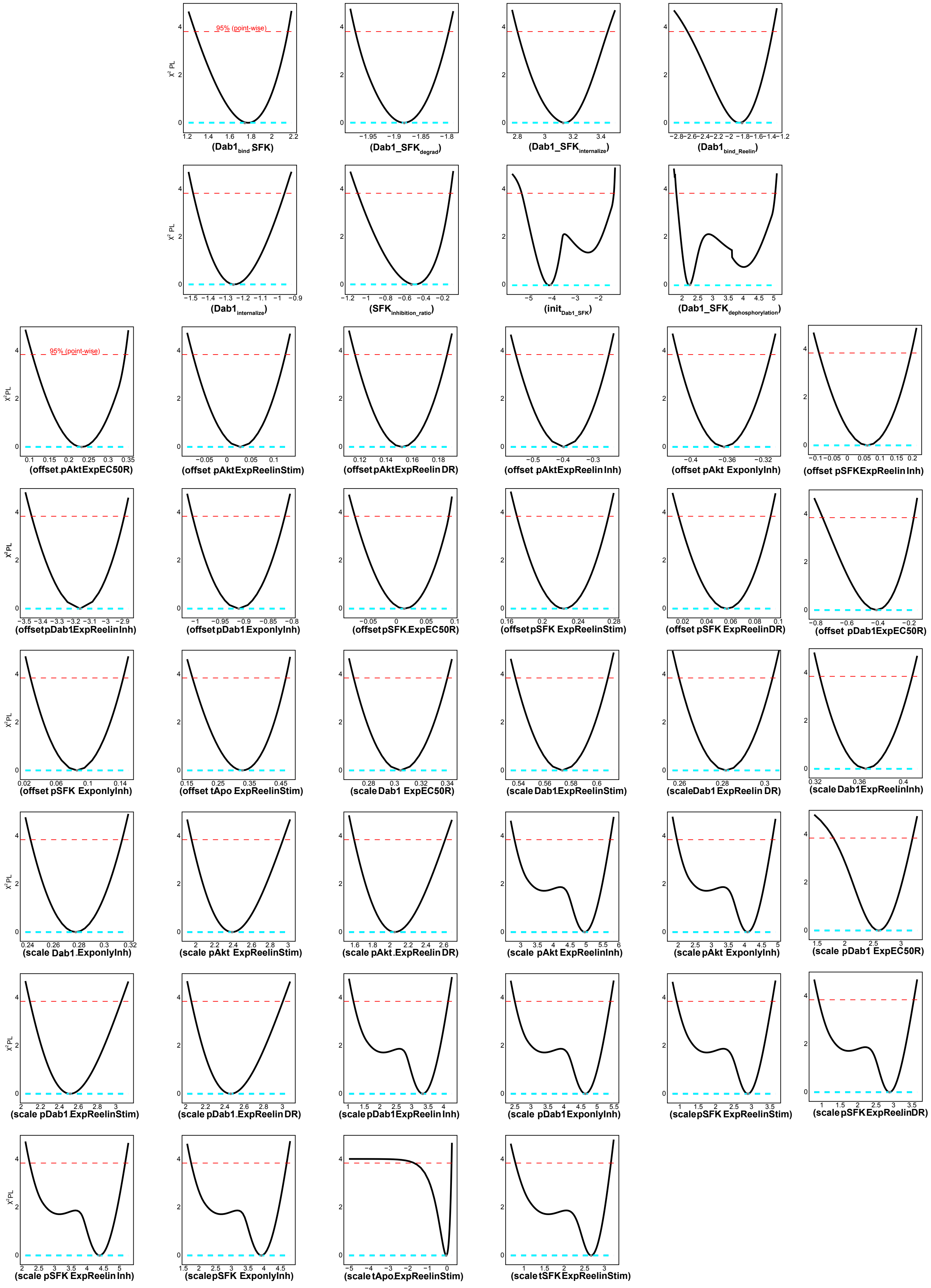

Fig S1: Parameter profiles of all model parameters of the complex model. Likelihood profiles of kinetic and observational parameters for all conditions after model reduction, which reach the 95 % threshold in both directions, thus are identifiable.
