# Supplementary material for: Mathematical model of early Reelin-induced Src family kinase-mediated signaling
Source: PLoS One. 2017 Oct 19;12(10):e0186927. doi: 10.1371/journal.pone.0186927 (PMC5648249; doi:10.1371/journal.pone.0186927)
Supplement: S1 Appendix — (PDF) [file pone.0186927.s002.pdf]

S1 Appendix

Mathematical model of early Reelin-induced Src family kinase-mediated signaling

Helge Hass, Friederike Kipkeew, Aziz Gauhar, Elisabeth Bouché, Petra May, Jens Timmer, Hans H. Bock

Description of Reelin model

Here, the properties and differential equations of the complex model used to describe the measurements are outlined (see Fig. 3 in the main text). To model Reelin-induced signaling, eight dynamic states are used in the model, summarized in **Table 1**. In this order, they describe the initial amount of Dab1 in the cytoplasm, of p-Dab1/SFK complex without and with bound SFK inhibitor, phosphorylated Dab1 after Reelin stimulation with and without bound SFK, the initial amount of Dab1 released from the receptor complex, and the initial amount of SFKs in the cytoplasm, respectively. Some initial concentrations are transformed according to analytically solved steady state equations [1]. These follow the assumption that the cell is in a steady state prior to ligand stimulation, thus

$$\dot{x}(t,\theta) \equiv 0 \qquad (6)$$

for all states given zero ligand concentrations, i.e. of Reelin and SFK inhibitor.

Table 1: Dynamic variables used in the complex model

Variable	Initial_Condition
Dab1	$Dab1\_SFK_{release}$ * [Dab1_SFK] * $Dab1\_SFK_{degrad}$/ ($Dab1\_SFK_{degrad}$ + $Dab1\_SFK_{dephosphorylation}$)
Dab1_SFK	[Dab1_SFK]
Dab1_SFKInh	0
$pDab1\_SFK_{\mathbf{Reelin}}$	0
$pDab1_{\mathbf{Reelin}}$	0
$Dab1\_SFKInh_{\mathbf{Reelin}}$	0

$Dab1_{\mathbf{released}}$	$Dab1\_SFK_{release}$ * [Dab1_SFK] / ($Dab1\_SFK_{degrad}$ + $Dab1\_SFK_{dephosphorylation}$)
SFK	$Dab1\_SFK_{release}$ * [Dab1_SFK] / ($Dab1_{bind\_SFK}$ * [Dab1])

The corresponding differential equations of the model states are given by:

$$\frac{d[Dab1]}{dt} = v_1 - v_2 - v_3 - v_8 - v_9 - v_{10} + v_{15} \tag{7}$$

$$\frac{d[Dab1\_SFK]}{dt} = v_2 - v_4 - v_6 \tag{8}$$

$$\frac{d[Dab1\_SFKInh]}{dt} = v_3 - v_5 - v_7 \tag{9}$$

$$\frac{d[pDab1_{Reelin}]}{dt} = v_8 - v_{12} \tag{10}$$

$$\frac{d[pDab1\_SFK_{Reelin}]}{dt} = v_6 + v_9 - v_{13} \tag{11}$$

$$\frac{d[Dab1\_SFKInh_{Reelin}]}{dt} = v_7 + v_{10} - v_{11} \tag{12}$$

$$\frac{d[Dab1_{released}]}{dt} = v_{12} + v_{13} - v_{14} - v_{15} \tag{13}$$

$$\frac{d[SFK]}{dt} = -v_2 - v_3 + v_4 + v_5 - v_9 - v_{10} + v_{11} + v_{13} \tag{14}$$

The flux expressions v_i of these equations, which determine the rate change of the model states, are provided in **Table 2**. Therein, v_1 describes Dab1 production. $v_{2,3}$ and $v_{9,10}$ describes interaction of Dab1 and SFKs, either basally or after Reelin stimulation, and with or without SFK inhibition. Unbinding of this complex is incorporated in $v_{5,11}$. Clustering of receptor-Dab1 or receptor-Dab1/SFK complexes through Reelin is included in $v_{6\text{-}8}$. Further, release of Dab1/SFK complexes or Dab1 from the receptor complex is described by $v_{4,12,13}$, with subsequent recycling or proteasomal degradation given by v_{15} and v_{14}, respectively.

Table 2: Model flux expressions

Flux	Flux expression
v_1	$Dab1_{prod}$
v_2	$[Dab1]* Dab1_{bind\_SFK} *[SFK]* (1- Inh *SFK_{inhibition\_ratio})$
v_3	$[Dab1]* Dab1_{bind\_SFK} *[SFK]* Inh *SFK_{inhibition\_ratio}$
v_4	$[Dab1\_SFK]*Dab1\_SFK_{release}$
v_5	$[Dab1\_SFKInh]*Dab1\_SFK_{unbind}$
v_6	$[Dab1\_SFK]* Dab1_{bind\_Reelin} * [Reelin\_input]$
v_7	$[Dab1\_SFKInh]* Dab1_{bind\_Reelin} * [Reelin\_input]$
v_8	$[Dab1]* Dab1_{bind\_Reelin}*[Dab1\_SFK_{Reelin}]*[Reelin\_input]$
v_9	$[Dab1]*[SFK] * Dab1_{bind\_SFK\_Reelin} *[Reelin\_input]*(1- Inh *SFK_{inhibition\_ratio})$
v_{10}	$[Dab1]*[SFK]*Dab1_{bind\_SFK\_Reelin} * [Reelin\_input]*Inh* SFK_{inhibition\_ratio}$
v_{11}	$[Dab1\_SFKInh_{Reelin}] * Dab1\_SFK_{unbind}$
v_{12}	$[pDab1_{Reelin}] * Dab1_{release}$
v_{13}	$[Dab1\_SFK_{Reelin}] * Dab1\_SFK_{release}$
v_{14}	$[Dab1_{Released}] * Dab1\_SFK_{degrad}$
v_{15}	$[Dab1_{Released}] * Dab1\_SFK_{recycle}$

The observation functions of the complex model, including scaling and offset parameters as well as the parameter perc_mes describing the fraction of inhibited SFKs bound to the receptor complex that are still phosphorylated at Y418, yet cannot trans-phosphorylate Dab1, are listed in Table 3. The experimental conditions used for model calibration, with their respective concentrations, time points and measured compounds are listed in **Table 4**.

Table 3: Observation functions of the complex model

Measurement	Observation function
tDab1	$scale\_Dab1 * (Dab1 + Dab1\_SFK + pDab1_{Reelin} + Dab1\_SFK_{Reelin} + Dab1\_SFKInh + Dab1\_SFKInh_{Reelin} + Dab1_{Released}) + offset\_Dab1$
pDab1	$scale\_pDab1 * (pDab1_{Reelin} + Dab1\_SFK_{Reelin} + Dab1\_SFK) + offset\_pDab1$
pAKT	$scale\_pAkt * (pDab1_{Reelin} + Dab1\_SFK_{Reelin} + Dab1\_SFK) + offset\_pAkt$
pSFK	$scale\_pSFK * (Dab1\_SFK + Dab1\_SFK_{Reelin} + perc\_mes * Dab1\_SFKInh_{Reelin}) + offset\_pSFK$
tSFK	$scale\_tSFK * (Dab1\_SFK + Dab1\_SFK_{Reelin} + SFK + Dab1\_SFKInh + Dab1\_SFKInh_{Reelin}) + offset\_tSFK$
tApoER2	$scale\_tApo\_ * (Dab1 + Dab1\_SFK + pDab1_{Reelin} + Dab1\_SFK_{Reelin} + Dab1\_SFKInh + Dab1\_SFKInh_{Reelin} + Dab1_{Released}) + offset\_tApo$

Summarizing, the model consists of 221 data points, 9 dynamical and 52 observational parameters.

In contrast, the monomer model (see Fig. 3 of the main text) consists of 8 dynamical and 50 observational parameters. The Bayesian information criterion for the complex model is 557.61, whereas the monomer model results in 572.05. The difference of 14.44 poses strong evidence for the complex model.

Since analytical solutions of non-linear ODE systems are in general not available, a numerical integration has to be performed. In this work, the dynamic system and its sensitivities were integrated by a multi-threaded implementation of the CVODES integrator [2,3]. Therein, an implicit backward differentiation formula (BDF) integration method [4] was chosen. The inner derivatives of the likelihood required for gradient-based parameter estimation were computed via analytically supplied forward sensitivities [5]. Numerical optimization was conducted using a trust-region based, large scale nonlinear optimization algorithm implemented in the MATLAB function LSQNONLIN [6]. For the mathematical modeling and visualization, the open-source and freely available framework Data2Dynamics [7], based on MATLAB, was used.

Table 4: Detailed listing of performed experiments

**Experiment**	Reelin stimulation	Dose-response of Reelin	Reelin stimulation with EC50 dose	Pan-SFK inhibition	Reelin stimulation after pan-SFK inhibition	Vldlr or ApoER2 knockout
**Stimulus**	**Saturating Reelin concentration**	**Dose-response measurement of Reelin**	**Reelin EC50 dose, 1% of saturating dose**	**Pan-SFK inhibitor**	**Reelin in saturating concentration after 30 min SFK inhibition**	**Saturating Reelin concentration**
**Measurement times**	Two time courses, with 8 time points between either 0-30 min or 0-240 min	0.01% to 100% of saturating Reelin concentration in 14 steps, measured at t=10 min	Eight time points up to 240 min	Six time points up to 240 min	Eight time points up to 240 min	Four time points up to 60 min
**Measured components**	Total Dab1, total SFK, total ApoER2, pDab1, pSFK, pAkt	Total Dab1, pDab1, pAkt	Total Dab1, pDab1, pSFK, pAkt	Total Dab1, pDab1, pSFK, pAkt	Total Dab1, pDab1, pSFK, pAkt	pDab1
**# of independent experiments on primary neurons, measured by WB**	28	6	4	6	6	4

References

1. Rosenblatt M, Timmer J, Kaschek D. Customized steady-state constraints for parameter estimation in non-linear ordinary differential equation models. Front Cell Dev Biol. 2016;4: 41.

2. Serban R, Hindmarsh AC. CVODES: The sensitivity-enabled ODE solver in SUNDIALS. In: ASME 2005 International Design Engineering Technical Conferences and Computers and Information in Engineering Conference. 2005. p. 257–69.

3. Hindmarsh AC, Brown PN, Grant KE, Lee SL, Serban R, Shumaker DE, et al. SUNDIALS: Suite of nonlinear and differential/algebraic equation solvers. ACM Trans Math Softw. 2005;31(3): 363–96.

4. Gear CW. The automatic integration of ordinary differential equations. Commun ACM. 1971;14(3): 176–9.

5. Leis JR, Kramer MA. The simultaneous solution and sensitivity analysis of systems described by ordinary differential equations. ACM Trans Math Softw. 1988;14(1): 45–60.

6. Coleman TF, Li Y. An Interior Trust Region Approach for Nonlinear Minimization Subject to Bounds. SIAM J Optim. 1996;6(2): 418–45.

7. Raue A, Steiert B, Schelker M, Kreutz C, Maiwald T, Hass H, et al. Data2Dynamics: A modeling environment tailored to parameter estimation in dynamical systems. Bioinformatics. 2015;31(21): 3558–60.
